# The 6-kilodalton peptide 1 of the family *Potyviridae*: small in size but powerful in function

**DOI:** 10.3389/fmicb.2025.1605199

**Published:** 2025-06-05

**Authors:** Liansheng Yu, Xayvangye Korxeelor, Ziyi Wang, Shuaifang Chang, Xue Jiang, Xiaoyun Wu, Xiaofei Cheng

**Affiliations:** ^1^College of Plant Protection, Northeast Agricultural University, Harbin, China; ^2^State Key Laboratory of Green Pesticide, Key Laboratory of Green Pesticide and Agricultural Bioengineering, Ministry of Education, Guizhou University, Guiyang, China

**Keywords:** 6K1, *Potyviridae*, viroporin, viral replication complex, membrane remodeling, antiviral targets

## Abstract

The *Potyviridae* family is one of the most economically significant groups of plant RNA viruses, causing severe yield losses in agriculturally important crops. Among the viral proteins encoded by potyviruses, the 6-kilodalton peptide 1 (6K1) has emerged as a critical, albeit poorly understood player in viral pathogenesis. Despite its small size, 6K1 exhibits diverse functions, including facilitating the assembly of viral replication complex (VRC), altering host membrane permeability as a viroporin, and interacting with host factors to promote infection. This review synthesizes current knowledge on 6K1, focusing on its structural characteristics, evolutionary conservation, molecular interactions, and potential as a target for antiviral strategies. We further discuss unresolved questions surrounding its putative ion channel activity, polyprotein processing dynamics, and functional parallels with animal virus viroporins. Understanding 6K1’s multifunctionality provides new insights into viral infection mechanisms and opens avenues for novel disease control approaches.

## 1 Introduction

Viral diseases represent a major threat to global food security and sustainable agriculture. The *Potyviridae* family is the largest family of plant RNA viruses, comprising at least 228 species grouped into 12 genera (Martínez-Turiño and García, 2020; Yang et al., 2021; Jaramillo-Mesa and Rakotondrafara, 2023). Viruses in the largest genus, *Potyvirus*, such as potato virus Y (PVY; *Potyvirus yituberosi*), soybean mosaic virus (SMV; *P. glycitessellati*), tobacco etch virus (TEV; *P. nicotianainsculpentis*), bean common mosaic virus (BCMV; *P. phaseoli*), maize dwarf mosaic virus (MDMV; *P. zaenanus*), turnip mosaic virus (TuMV; *P. rapae*), plum pox virus (PPV; *P. plumpoxi*), sweet potato feathery mottle virus (SPFMV; *P. batataplumei*), and sugarcane mosaic virus (SCMV; *P. sacchari*), which cause significant economic yield losses in crops of the families *Solanaceae*, *Leguminosae*, and *Chenopodiaceae* worldwide. For instance, PVY alone can reduce potato yields by 20–80% during severe outbreaks (Yang et al., 2021; Wani et al., 2023; Liu et al., 2023; Belabess et al., 2024; Kumar and Dasgupta, 2024; Mardanova et al., 2024).

Members of the *Potyviridae* family (potyvirids) induce a range of symptoms that affect plant growth and yield, including leaf shrinkage, necrosis, mottling, yellowing, and plant stunting. They also reduce the quality of fruits and tubers and increase susceptibility to other phytopathogens (Yang et al., 2021; Luan et al., 2024; Qin et al., 2024; Yang et al., 2024; Dupuis et al., 2024; Kamran et al., 2025). In addition, virus infection compromises the storage quality of potatoes, imposing an economic burden on farmers (Yang et al., 2021; Kamran et al., 2025; Dupuis et al., 2024). The severity of potyviral infection is exacerbated by their diverse transmission modes, including aphids, mechanical fiction, fungus, and seed, which complicates efforts for disease prevention and control (Crosslin, 2013; Gadhave et al., 2020; Bhoi et al., 2022). Therefore, understanding the biological characteristics and mechanisms of their pathogenesis are crucial to developing effective prevention and control strategies.

The genome of potyvirids consists of one or two positive-sense single-stranded RNAs (+ssRNA) of approximately 10 kilobases (kb) in length in total, that is covalently linked to VPg at the 5′ end and polyadenylated at the 3′ end (Yang et al., 2021). Each genomic +ssRNA encodes a long open reading frame (ORF) that is translated into a polypeptide. Typically, potyvirids encode 10 multifunctional proteins, namely, First Protein (P1), Helper Component-Proteinase (HC-Pro), Third Protein (P3), 6-kilodalton peptide 1 (6K1), Cylindrical Inclusion (CI), 6-kilodalton peptide 1 (6K2), Viral Protein Genome-linked (VPg), Nuclear Inclusion a-Protease (NIa-Pro), Nuclear Inclusion b (NIb), and Coat Protein (CP) (Yang et al., 2021; Hýsková et al., 2024). In addition, all potyvirids contain an RNA polymerase slippage motif within P3 cistron, enabling the expression of an additional polypeptide that is cleaved into P1, HC-Pro, and P3 N-terminal fused with Pretty Interesting *Potyviridae* ORF (P3N-PIPO). A few sweet-potato-infecting potyvirids also possess a similar slippage motif within the P1 cistron, leading to the translation of an additional protein, P1 N-terminus fused with the Pretty Interesting Sweet Potato potyviral ORF (P1N-PISPO) (Yang et al., 2021; Jaramillo-Mesa and Rakotondrafara, 2023; Valli et al., 2024). Emerging evidence suggests that the complementary RNA strand of *Potyviridae* has protein-coding capacity, challenging the traditional view of their unidirectional genomic expression and enriching our understanding of viral coding potential and pathogenicity (Gong et al., 2021; Gong et al., 2023; Li et al., 2024; Gong et al., 2025). Among these proteins, 6K1 remains one of the least understood viral proteins. It is highly conserved in potyvirids and located between P3 and CI cistrons (Cui and Wang, 2016; Hu et al., 2023a,b). Recent studies reveal that 6K1 functions as a viroporin, which plays a critical role in viral infection. This discovery provides new insights into the molecular mechanisms of potyviral infection (Chai et al., 2024). This review summarized the structural and functional properties of 6K1, discussed its role in viral replication and membrane remodeling, explored interactions with host factors and immune evasion strategies, evaluated evolutionary adaptations across *Potyviridae*, and assessed potential antiviral strategies targeting 6K1.

## 2 Structural and biochemical properties of 6K1

### 2.1 Protein characteristics and subcellular localization

The 6K1 protein is located between the P3 and CI cistrons in all known potyvirids with sizes ranging from 6 to 7 kilodalton (kDa). Bioinformatics analyses suggest that 6K1 contains two transmembrane helices (TMH1 and TMH2) (Chai et al., 2024). TMH1 is highly hydrophobic, and is primarily composed of the amino acids Ser, Glu, Ile, Phe, Val, and Phe, while TMH2 is less hydrophobic and contains several highly conserved basic residues, e.g., His and Lys, which comprises a so-called K/R-rich motif (Chai et al., 2024; Hu et al., 2025). Alphafold-assistant modeling suggests that the two transmembrane helices adopt a helix-turn-helix structure. The cleavage site between P3 and 6K1 in many, if not all potyvirids, is not favor for NIa-Pro cleavage, leading to the coexistence of free 6K1 and P3-6K1 fusion proteins in virus-infected cells, which highlights the functional versatility of 6K1 in viral infection (Riechmann et al., 1995; Cui and Wang, 2016).

It is widely accepted that 6K1, together with P3 and 6K2, constitutes three potyviral membrane-associated proteins (Cui and Wang, 2016; Bera et al., 2022; Hu et al., 2025). Immunogold labeling using polyclonal antiserum revealed that soybean mosaic virus (SMV)-encoded 6K1 predominately localizes at the cell periphery (Hu et al., 2023a,b). Transient expression of 6K1 from TuMV and PPV as C-terminal YFP-tagged recombinant proteins in *Nicotiana benthamiana* ephemeral cell showed cytosolic and nuclear localization, while N-terminal YFP-tagged TuMV 6K1 displayed membrane-associated subcellular distribution and colocalized with the endoplasmic reticulum (ER) network. By contrast, P3-6K1 fusion protein appeared as small granules localized to the ER network (Riechmann et al., 1995; Cui and Wang, 2016; Chai et al., 2024; Hu et al., 2025). In the context of virus infection, the subcellular localized of C-terminal YFP-tagged PPV 6K1 was analyzed by expressing an additional copy between P1 and HC-Pro (Riechmann et al., 1995; Cui and Wang, 2016; Chai et al., 2024; Hu et al., 2025). Results showed that PPV 6K1 forms punctuate membrane-associated granules and colocalizes with viral replication complexes (VRCs) adjacent to chloroplasts. These findings underscore the multifunctional role of 6K1 in viral life cycle and suggest that it serve as critical regulatory hubs for coordinating viral processes (Cui and Wang, 2016).

### 2.2 Function as a viroporin

**Figure 1:**
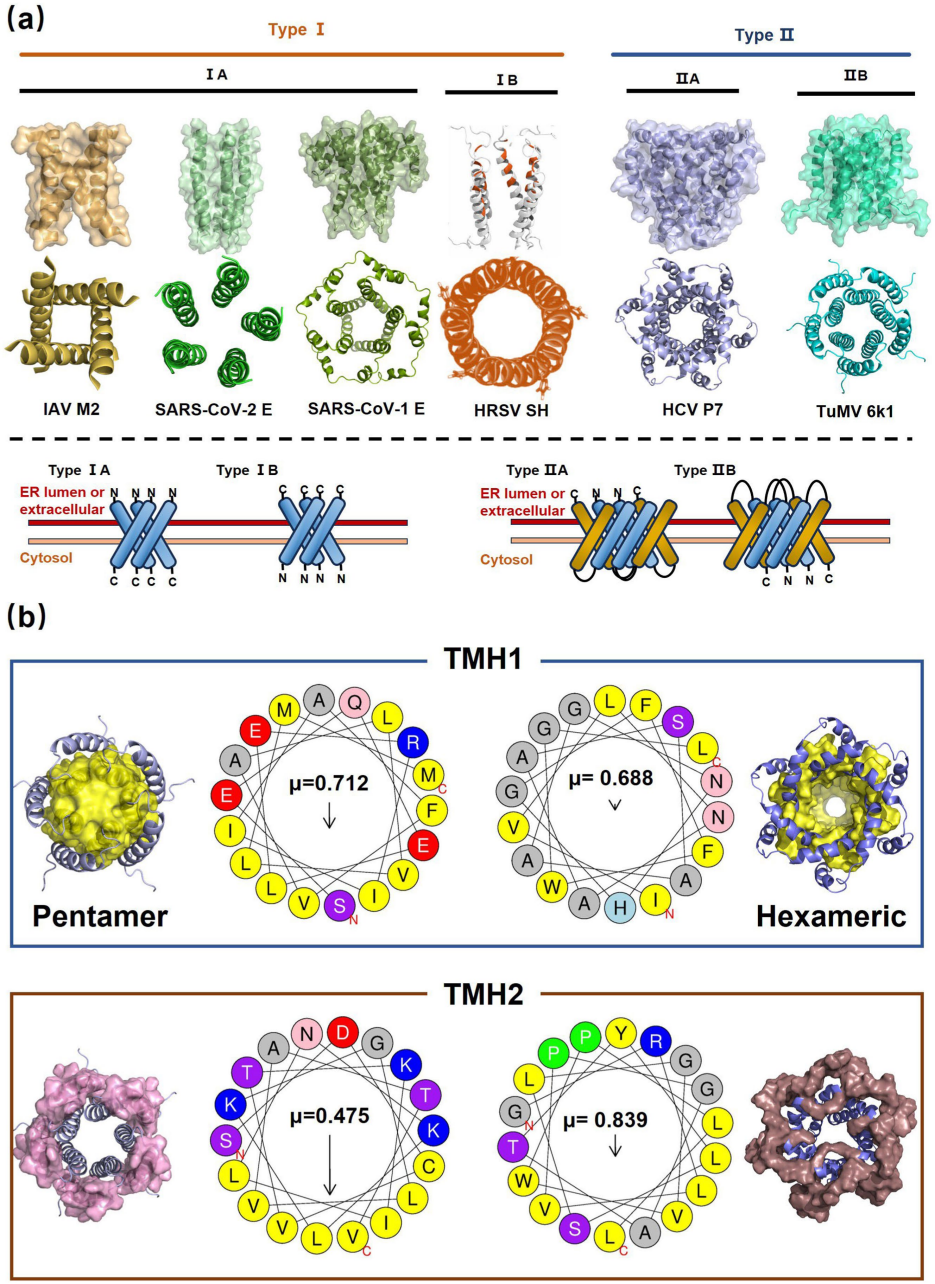
The structure of viroporins. **(a)** Schematic classification of viroporins. IA and IB viroporins contain one TMH with the C terminus in the cytosol and endoplasmic reticulum (ER) lumen or extracellular, respectively; IIA and IIB viroporins contain two TMHs with both N- and C-termini in the cytosol and ER lumen or extracellular, respectively. Type III viroporins contain three TMHs and are not illustrated due to lack of structure; Influenza virus A M2 (IAV M2; PDB ID: 4QKM); Syndrome coronavirus 2 E peptide (SARS-COV-2 E; PDB ID: 8SUZ); Syndrome coronavirus 1 E peptide (SARS-COV-1 E; PDB ID: 5 × 29);Human respiratory syncytial virus SH (HRSV SH); Hepatitis C virus P7 (HCV P7; PDB ID: 2M6X); **(b)** Structural comparison of TuMV 6K1 and HCV P7. The helical wheels showing TMH 1 (top panel) and TMH 2 (top panel) of the putative pentamer of TuMV 6K1 and HCV P7 hexamer, generated using HeliQuest (numbers indicate the hydrophobic moment). The cartoon views of the TMH 1 (top panel) and TMH 2 (top panel) of the putative pentamer of TuMV 6K1 and HCV P7 hexamer are also shown.

Alphafold–assisted structure modeling and biochemical assays suggest that TuMV and PVY 6K1 forms pentamers with a central hydrophobic cavity, resembling viroporins, a specialized group of virus-encoded small ion channels (Chai et al., 2024). Decades ago, researchers observed increased permeability to ions and small molecules of cells infected with animal viruses, but it was not until 1995 that Louis Carrasco coined the term “viroporins” to describe viral proteins with ion channel activity (Carrasco, 1995). According to the classification criteria proposed by Devantier et al. (2024a,b) and Nieva et al. (2012), viroporins are divided into two major classes (I and II) based on the number of TMHs) (Figure 1a). Furthermore, depending on the cytoplasmic and organellar accessibility of their N- and C-terminal domains, viroporins are further divided into two subtypes A and B (Pielak and Chou, 2011; Nieva et al., 2012; Devantier et al., 2024a,b; Gebert et al., 2024). Recently, a class III viroporin category has been proposed (Pielak and Chou, 2011; Nieva et al., 2012; Devantier et al., 2024a,b; Gebert et al., 2024). Structural predictions of the TuMV 6K1 pentamer indicate that 6K1 adopts a type II viroporins classification, characterized by two TMHs. In addition, a topology assay demonstrated that the N-terminus of 6K1 is exposed to the cytosol (Chai et al., 2024), confirming that 6K1 belongs to the class IIB subtype viroporins.

**Figure 2:**
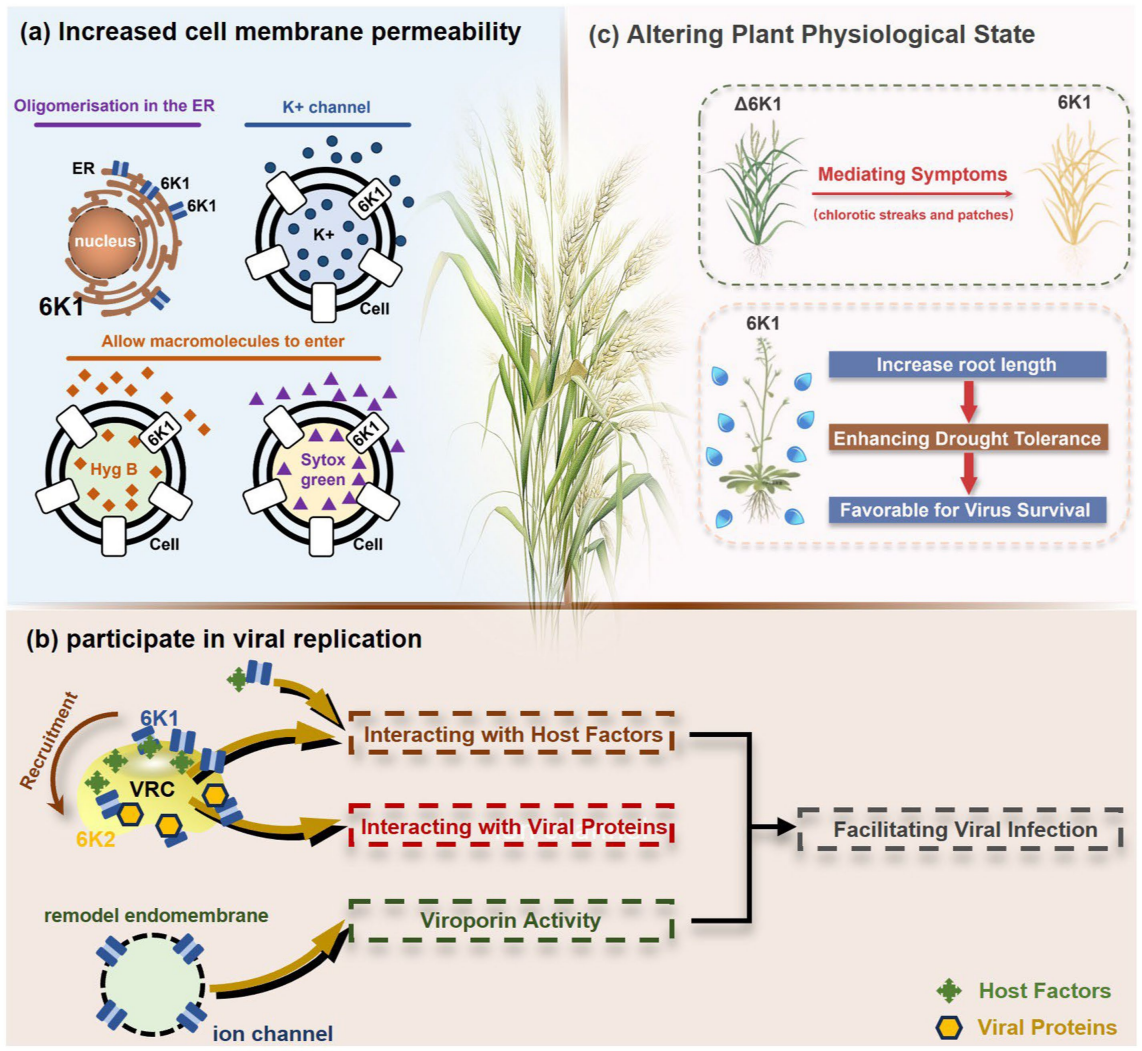
The illustration of 6K1 function during viral infection. **(a)** 6K1 localizes to ER and form oligomers. It increases membrane permeability, enabling the uptake of Hygromycin B and Sytox Green and enhances potassium ion flux. **(b)** 6K1 is recruited to VRCs to facilitate viral infection possibly via interacting with host and viral proteins and its viroporin activity. **(c)** 6K1 adds in viral symptom development, promotes root elongation and improves drought tolerance.

TuMV 6K1 forms oligomers via self-interaction on the ER and can enhances membrane permeability in *Escherichia coli* and *N. benthamiana*. Additionally, it complements the growth of yeast mutants deficient in potassium ion channels on low-potassium medium (Chai et al., 2024; Gao et al., 2024). In addition to TuMV 6K1, other potyviral 6K1 proteins and their cognate 7K counterparts similarly alter membrane permeability, exhibiting toxicity in *E. coli* (Chai et al., 2024; Gao et al., 2024). Notably, both TuMV 6K1 and hepatitis C virus (HCV) P7 facilitate the uptake of the macromolecular dye Sytox green when expressed in *N. benthamiana* leaves (Figure 2a) (Chai et al., 2024; Gao et al., 2024), confirming their role in modulating membrane permeability. Viroporins exhibit significant diversity in amino acid composition and ionic selectivity. For instance, severe acute respiratory syndrome coronavirus-1 (SARS-CoV-1) E, HCV P7, human immunodeficiency virus-1 Vpu (HIV1 Vpu), and ross river virus 6K (RRV 6K) display high selectivity for Na^+^ and K^+^, but low affinity for Cl^−^ (Melton et al., 2002; Premkumar et al., 2004; Wilson et al., 2004; Surya and Torres, 2022; Surya et al., 2023). SARS-CoV-1 E shows a 5- to 10-fold preference for Na^+^ over K^+^ and an 10-fold preference for K^+^ over Cl^−^ (Surya et al., 2023; Surya and Torres, 2022; Wilson et al., 2004; Melton et al., 2002). IAV M2 is proton-selective, mediating virion acidification, viral envelope-organelle membrane fusion, and cytoplasmic virion release (Lakadamyali et al., 2003; Xia et al., 2022). TuMV 6K1 and barley yellow striate mosaic virus (BYSMV; *Cytorhabdovirus hordei*) P7 also exhibit K^+^-selective permeability (Chai et al., 2024; Gao et al., 2024). Despite these functional parallels, potyviral 6K1 proteins share no detectable amino acid sequence homology with known viroporins. The specific ion selectivity of potyviral 6K1 remain unresolved, primarily due to the absence of electrophysiological evidence.

## 3 Functional roles of 6K1 in viral infection

### 3.1 Coordinates VRC assembly and participates in replication

6K1 plays a central role in viral replication (Figure 2b) (Chai et al., 2024; Cui and Wang, 2016; Cui and Wang, 2019). In the early stages of viral infection, 6K1 of PPV forms punctate structures and colocalizes with VRCs induced by 6K2 from ER (Cui and Wang, 2016). During tobacco vein banding mosaic virus (TVBMV) infection, 6K2 recruits 6K1 to the VRCs near the chloroplasts, where it colocalizes with NIb, the viral RNA-dependent RNA polymerase (RdRp) (Geng et al., 2017). Deletion or functional defects of pepper veinal mottle virus (PVMV; *P. capsivenae*)-encoded 6K1 result in a substantial reduction in viral replication (Hu et al., 2025). Recent studies on PPV infection have found that 6K1, 6K2, and NIb colocalize and jointly coordinate the assembly of VRCs during the early stages of infection (Cui and Wang, 2016). Importantly, disruption of the N- or C-terminal cleavage sites of 6K1 prevents its proteolytic release from the viral polyprotein precursor, thereby leading to partial or complete inhibition of viral replication, suggesting the crucial role of 6K1 in the viral life cycle (Cui and Wang, 2016). Moreover, a series of deletion analyses of PPV 6K1 have demonstrated that the removal of any internal short sequence, truncation of the extension region (even when the conserved cleavage sites are retained), or complete deletion of the 6K1-encoding sequence results in attenuated viral replication. These findings suggest that 6K1 has stringent spatiotemporal requirements for coordinating viral replication dynamics (Cui and Wang, 2016). However, the precise role of 6K1 in viral replication remains poorly understood. One possibility is that 6K1 is directly involved in the assembly of VRCs (Bera et al., 2022; Chai et al., 2024; Cui and Wang, 2016; Fang et al., 2024). Alternatively, 6K1 may affect the spatial organization or stability of VRCs. Additionally, 6K1 can interact with other viral proteins, which may affect VRCs assembly and viral replication (Fang et al., 2024; Morozov and Solovyev, 2020; Wei and Wang, 2008; Xue et al., 2023). Another hypothesis is that the function of 6K1 viroporin activity may contribute to viral replication by modulating the pH or ion homeostasis in VRCs. Nevertheless, further investigations are needed to fully illustrate these possibilities.

### 3.2 Viroporin activity modulates viral replication

Many viral membrane proteins remodel endomembrane for viral replication or movement. For instance, 6K2 can remodel ER into vesicles to host viral replication (Bera et al., 2022; Cui and Wang, 2016; Hu et al., 2023a,b). Similarly, 6K1 also exhibits endomembrane-remodeling activity: transiently expressed TuMV 6K1 forms granules of various sizes on the ER (Chai et al., 2024). Confocal microscopy observations revealed that PPV 6K1 forms punctate inclusion during viral infection when expressed as a 6K1-GFP fusion protein between the P1 and HC-Pro cistron (Cui and Wang, 2016). Notably, the P3-6K1 fusion protein, along with other potential 6K1-containing complexes, may play distinct yet complementary roles in viral replication, membrane remodeling, and intracellular trafficking, thereby contributing to the establishment of VRCs and systemic viral spread.

### 3.3 Autophagy-mediated degradation orchestrates viral replication

The accumulation of 6K1 is extremely low in late viral infection stages, which is closely linked to its targeted degradation by the host autophagy system (Hu et al., 2025). For instance, treatment with the E-64d inhibitor or silencing of the autophagy gene *N. benthamiana Autophagy-related Protein 7* (*NbATG7*) has been shown to enhance the stability of 6K1 while concomitantly delaying systemic viral infection (Hu et al., 2025). This paradoxical phenomenon suggests that the degradation of 6K1 may represent an active viral strategy to co-degrade host antiviral factors, thereby indirectly promoting viral replication. Alanine scanning mutagenesis has revealed that the K/R-rich motif is critical for its autophagy-mediated degradation of 6K1: mutants (e.g., V32A or K34A) that evade autophagy recognition exhibit delayed viral spread in the host (Hu et al., 2025). A similar mechanism has also been demonstrated in TuMV, where 6K1 degradation depends on its interaction with the autophagy receptor ATG8; disruption of this process significantly reduces viral replication (Bera et al., 2022; Hu et al., 2025). These finding suggest that the 6K1 orchestrates viral replication through a dual mechanism: firstly, it serves as a structural component of the replication complex to directly facilitate viral RNA synthesis; secondly, it may simultaneously coordinate the elimination of host defense factors through dynamic regulation of its own abundance through autophagy-mediated degradation. This sophisticated “synthesis-degradation” regulatory paradigm not only optimizes viral resource utilization but also underscores the evolutionary adaptability of 6K1 in host-pathogen interactions, revealing new therapeutic targets for antiviral intervention.

## 4 Host-virus interactions

### 4.1 Subversion of host defense

In recent years, accumulating evidence has demonstrated that 6K1 establishes dynamic interaction networks with both host factors and viral proteins to optimize the microenvironment for viral replication and facilitate viral proliferation (Figure 2b) (Cui and Wang, 2016; Bera et al., 2022; Hu et al., 2023a,b; Tatineni et al., 2023; Fang et al., 2024; Hu et al., 2025). Soybean 40S ribosomal protein S8 (GmRPS8), an essential ribosomal component, interacts with 6K1 in yeast-two-hybrid (Y2H) and bimolecular fluorescence complementation (BiFC) assays, play a crucial role in SMV infection (Hu et al., 2023a,b). During TVBMV infection, 6K2 recruits 6K1 to VRCs, where 6K1 interacts with both 6K2 and NbPsbO1, a component of the PSII oxygen-evolving complex, to participate in viral replication (Geng et al., 2017). 6K1 competitively binds to Nb14-3-3 h, a key plant defense protein, disrupting its interaction with *N. benthamiana* Translationally Controlled Tumor Protein (NbTCTP) to promote PVY infection (Fang et al., 2024). When ectopic expression of TuMV 6K1 in *N. benthamiana*, it reduces transcripts related to jasmonic acid biosynthesis and cysteine proteinase inhibitors while enhancing TuMV accumulation in systemic leaves (Bera et al., 2022). However, whether 6K1 helps viruses evade host immune system by suppressing RNA silencing or interfering with other antiviral defense mechanisms, and whether 6K1 modulates host immune responses through interactions with host immune factors (such as products of R genes), require further investigations.

### 4.2 Symptom development

Studies have shown that co-expression of 6K1, 6K2, and NIa-Pro in *A. thaliana* significantly improves drought tolerance, and *A. thaliana* overexpressing 6K1 shows notably greater root length compared to those expressing NIa-Pro or 6K2 (Prakash et al., 2023). This drought tolerance mechanism benefits both virus and host by increasing host survival rate under drought conditions and therefore extending the viral replication and transmission window (Prakash et al., 2023). Heterologous expression of wheat streak mosaic virus (WSMV; *Tritimovirus tritici*) induces characteristic viral symptoms, including severe chlorotic streaks and leaf spotting, which mirrors symptoms seen during natural WSWV infection (Tatineni et al., 2023). This dual functionality of 6K1, enhancing host stress tolerance while promoting viral pathogenesis, highlights its sophisticated role in virus-host interactions. The physiological modifications induced by 6K1 create an environment that simultaneously sustains host viability while facilitating viral proliferation and symptom development (Figure 2c).

## 5 Evolutionary adaptations

### 5.1 Evolution of 6K1: dual-driven by both host and vector pressures

**Figure 3:**
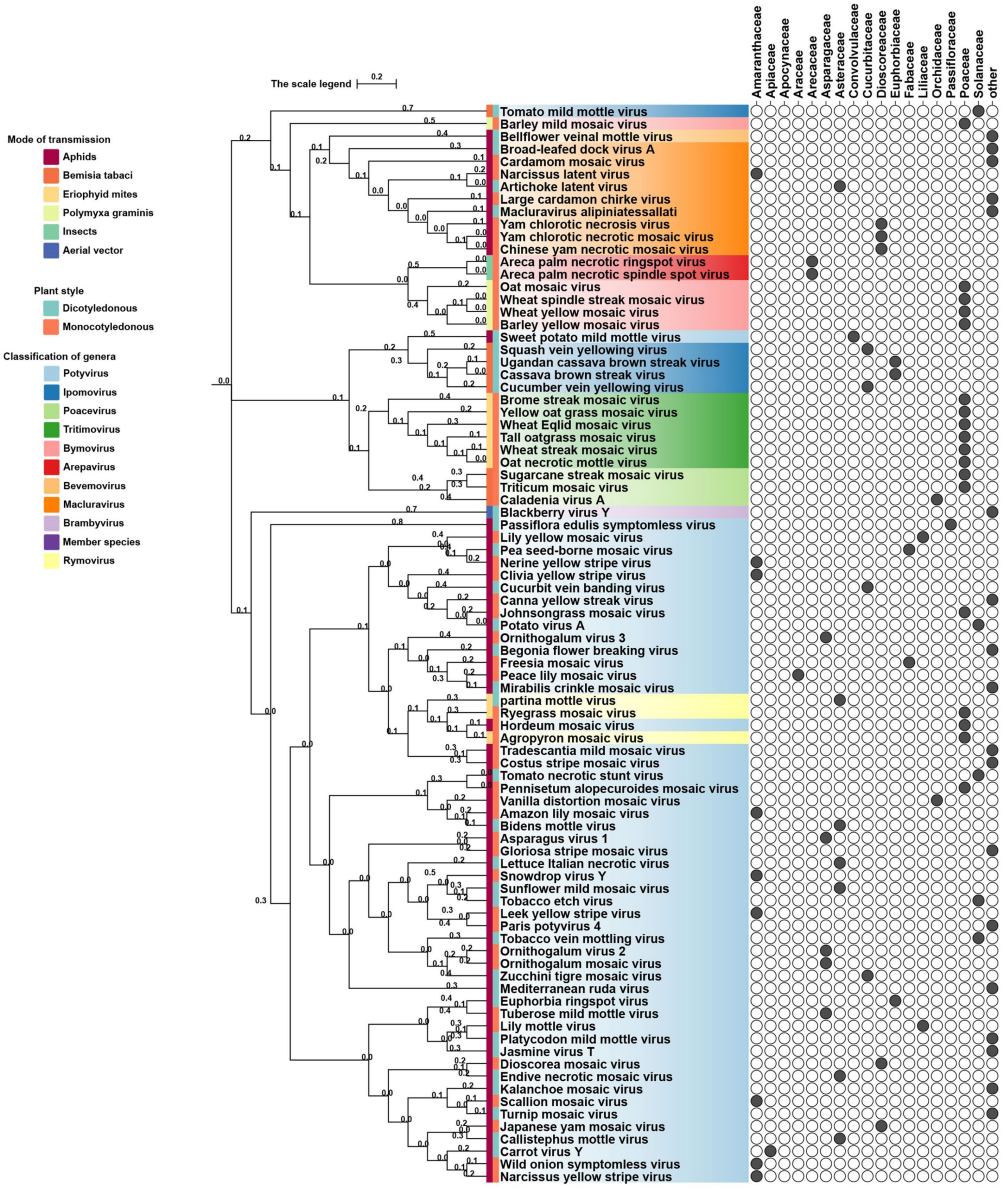
Phylogeny of potyviral 6K1 and its 7K homolog proteins. The tree was constructed using the Neighbor-Joiningmethod and the numbers on branches indicate the bootstrap supporting values. The transmission manner, host specificity, and taxonomy of each virus were indicated. Full information of the tree can be found in the Supplementary Figure 1.

Phylogenetic analyses reveal both vector-driven clustering patterns and host-specific lineage diversification of potyviral 6K1 and its 7K homologs. For instance, aphid-transmitted genera (*Potyvirus* and *Macluravirus*) and eriophyid mite-transmitted genus (*Tritimovirus*) form well-supported distinct monophyletic clades. *Rymovirus* members (e.g., parthenium mottle virus, ryegrass mosaic virus, and agropyron mosaic virus) exhibit evolutionary convergence with potyviruses, while sweet potato mild mottle virus (*Potyvirus*) shows atypical clustering with ipomoviruses (e.g., Squash vein yellowing virus), suggesting ecological niche adaptation. *Poacevirus* and *Tritimovirus* (*Poaceae* specialists) share close genetic relationship, whereas *Arepavirus* (*Arecaceae* specialists) and *Bymovirus* (*Poaceae* specialists) form discrete host-adapted clusters. *Potyvirus*, the largest genus with broad host range spanning monocots and dicots, displays extensive intragenus differentiation, reflecting clear patterns of adaptive radiation across monocot/dicot hosts. For instance, bermuda grass southern mosaic virus clusters with *Amaranthaceae* and *Poaceae*-infecting lineages infecting. These complex evolutionary patterns of potyviral 6K1 suggesting selective pressures from both hosts and transmission vectors, such as host antiviral RNA silencing, crop-specific protein adaptations, and vector transmission efficiency optimization. Such pressures result in a unique evolutionary balance between strict conservation of replication-critical domains and adaptive plasticity in host/vector-interacting regions. This evolutionary framework highlights 6K1’s dual evolutionary strategy - maintaining core functional elements while acquiring specialized adaptations to diverse ecological niches (Figure 3) (Xie et al., 2023).

### 5.2 Core residue stability vs. vector-specific adaptation

**Figure 4:**
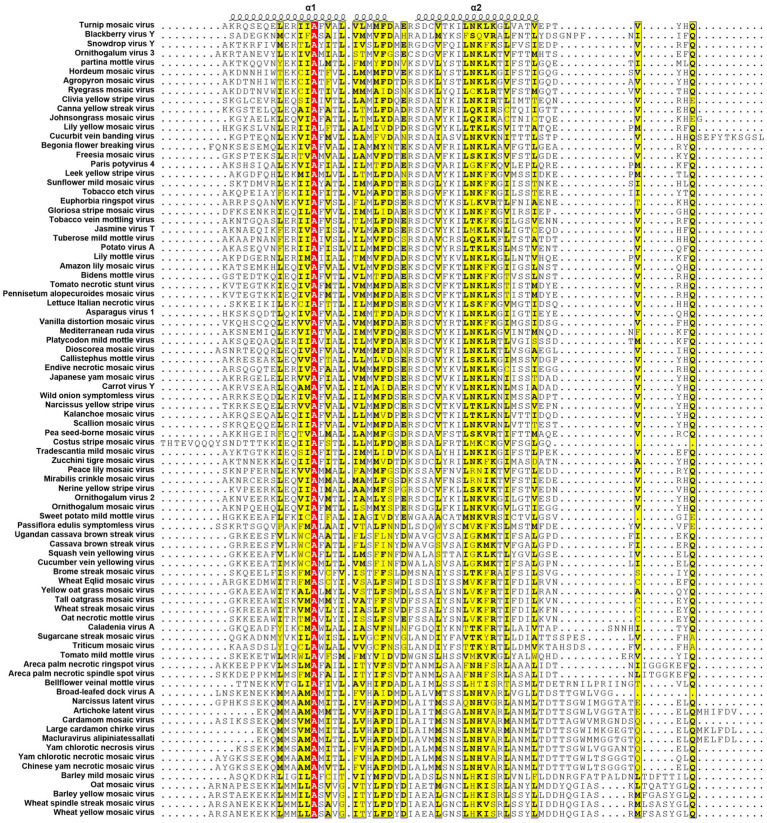
Multiple sequence alignment of potyviral 6K1 and its 7K homologs. The alignment was produced using the Clustal X software and presented using ESPript. The completely and functional conserved residues are shown in red and yellow background, respectively. The full information of this figure can be found in the Supplementary Figure 2.

All known potyvirids contain 6K1 or its 7 K homolog between the P3 and CI cistrons. Bioinformatics analyses reveal two conserved TMHs in all 6K1 proteins, and the helix-turn-helix hairpin topology also is also consistently in 6K1 across potyvirids (Chai et al., 2024). Moreover, the three highly conserved Lys residues in 6K1 (Lys33, Lys37, and Lys39) that are critical for oligomerization are also functional conserved (Figure 4) (Chai et al., 2024; Cui and Wang, 2016). The “RSD” motif, which is highly conserved in potyviruses, have been replaced by “IAE” “LAL,” and “TAN” in viruses of the genera *Macluravirus*, *Bymovirus*, and *Tritimovirus*, respectively. These data highlight the crucial role of 6K1 in viral infection process with universal structural conservation, genus-specific functional adaptation, and essential conserved residues for oligomerization (Figure 4).

6K1 also display significant amino acid sequence and size diversity across potyvirids. The length of 6K1 proteins is typically 6 kDa in most potyvirids, while expands to 7 kDa in certain viruses of the genera *Macluravirus* and *Bymovirus*, which have been designated as 7K. Sequence alignment suggest that the 7K variants contain an 8–10 residues extension at the TMH2 C-terminus. The function of these amino acids is unknown at the present, although these residues have been proposed in interacting with viral or host proteins or regulating viroporin activity. Besides, the N- and C-terminal residues of 6K1 also show high variability. These two parts comprising the NIa-Pro recognition and cleavage site and thus likely co-evolved with NIa-Pro protease. These findings indicate that the 6K1 protein follows a dual evolutionary strategy to balance functional constraints and ecological adaptation. 6K1 of viruses infecting dicot plants tend to enrich acidic residues (Asp/Glu), while those of viruses infecting monocot plants prefer basic residues (Lys/Arg). Additionally, the 6K1 of dicot-infecting viruses maintain a hydrophobic transmembrane core (Leu/Val/Ile > 85%) and conserved Phe/Leu residues, while those of monocot-infecting viruses in genera *Tritimovirus* and *Bymovirus* accumulate Ser → Pro/Lys → Glu mutations in their transmembrane regions (Figure 4).

The 6K1 protein also exhibits evolutionary adaptations corresponding to different transmission pathways within the *Potyviridae* family. For instance, 6K1 of aphid-transmitted viruses exhibit C-terminal Pro/Gly enrichment and contain highly conserved Asp/Glu residues (mutation rate <3%), while that of mite-transmitted viruses show a higher frequency of Lys → Glu mutations in the transmembrane region (22%) and an increased Phe/Leu residue proportion, 6K1 of whitefly-transmitted viruses possess GRREESF sequence insertions and Val → Leu mutations, and that of fungus-transmitted viruses exhibit a “LNKLK” to “LNHV” motif alteration. These transmission-specific adaptations likely demonstrate how 6K1 has evolved unique molecular strategies to optimize vector-specific requirements, highlighting its remarkable evolutionary plasticity while maintaining core functions.

## 6 Antiviral strategies targeting 6K1

### 6.1 Small-molecule inhibitors

Traditional inhibitors primarily target viral RdRp or helicase, such as ribavirin, which disrupts the catalytic core of TSWV RdRp (Li et al., 2025; Wang et al., 2025), Z9 effectively inhibits co-condensate formation between TSWV nucleoprotein (N) and viral RNA, reducing viral proliferation (Zan et al., 2025). Recently, pH-sensitive (GPS) polymer nanoparticles have been found to selectively bind to infected cell membranes or viral envelopes, even envelope disruption (Sun et al., 2022). This leverages pH changes in infected cells as a therapeutic target. For viroporins, most inhibitors act via either physical block the channel or disrupt their oligomerisation. Although 6K1 have recently been confirmed function as a viroporin, research on specific inhibitors remains lacking. Despite lacking sequence homology, 6K1 adopts spatial folding and conformations analogous to IAV M2, HCV P7, SARS-CoV E1/E2 and HIV Vpu with a high hydrophobic central cavity (Figure 1b) (Devantier et al., 2024a,b; Elmasri et al., 2022; Surya et al., 2023; Surya and Torres, 2022). Therefore, it is possible that these inhibitors may also function for 6K1. For example, amantadine also inhibits BYSMV P9, another recently identified plant virus-encoded Class I viroporin (Gao et al., 2024). Table 1 listed the major viroporin inhibitors of viroporins. Among these inhibitors, Adamantane and Amiloride have significant inhibitory effects on IAV M2 and HCV P7, while Flavonoids, Emodin, and Tretinoin target SARS-CoV E1/E2. Thus, these compounds may function against 6K1 as well. Nevertheless, it is necessary to test the existing viroporin inhibitors against using the membrane permeability and electrophysiological assays and then rational optimize these compounds for higher specificity and potency against 6K1.

**Table 1:** Known inhibitors of viroporins.

Class	Compound	Pubchem CID	Structure	Target
Adamantane	Amantadine	2130	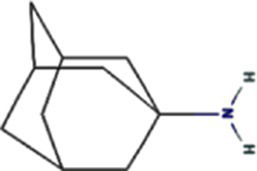	IAV M2 (Toft-Bertelsen et al., 2021) SARS-CoV-2 E (Ge, 2020) HCV P7 (Chen and Wang, 2024) BYSMV P9 (Gao et al., 2024)
	Memantine	4054	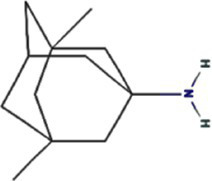	SARS-CoV-2 E (Jorge et al., 2024)
	Rimantadine	5071	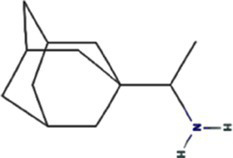	SARS-CoV-1 E (Breitinger et al., 2021) SARS-CoV-2 3a (Fam et al., 2023) HCV P7 (Devantier et al., 2024a,b) IAV M2 (Kumar and Sakharam, 2024) HPV E5 (Wetherill et al., 2012)
	Spiro	10398047	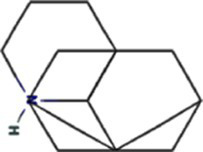	IAV M2 (Tietjen et al., 2024)
	Spiro-Adamantane	64599	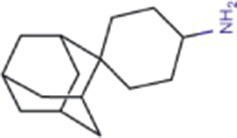	IAV M2 (Kumar and Sakharam, 2024)
Spirane-Amine	BL-1743	9837397	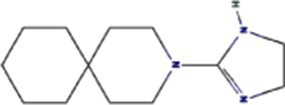	IAV M2 (Ginex and Luque, 2021)
Alkyl Imino-Sugar	NN-DNJ	501640	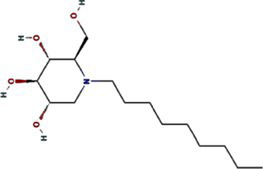	HCV P7 (Dahl et al., 2018) IAV M2 (Scott et al., 2020) Hepatitis B virus X (Lee et al., 2018)
Arenesulfonic acid	DIDS	5281951	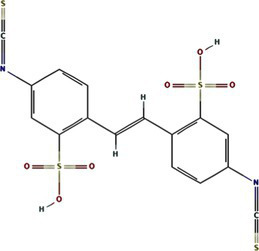	Influenza C virus CM2 (Hongo et al., 2004) Influenza D Virus DM2 (Kesinger et al., 2018) Enterovirus 71 2B (Xie et al., 2011)
Amiloride	HMA	1794	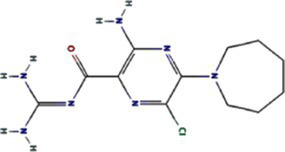	Human coronavirus E (Somberg et al., 2023) Murine hepatitis virus E (Roth-Cross et al., 2008) SARS-CoV-1 E (Jalily et al., 2022) SARS-CoV-2 E (Somberg et al., 2023) Dengue virus M (Premkumar et al., 2005) HCV P7 (Devantier et al., 2024a, 2024b) IAV M2 (Elmasri et al., 2022) HIV-1 VPU (Rosenberg et al., 2016)
	BIT225	12004418	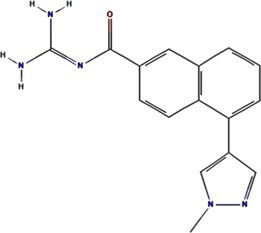	SARS-CoV 2 E (Ewart et al., 2023) HCV P7 (Ewart et al., 2023) HIV-1 VPU (Ewart et al., 2023)
Antibiotics	Doxycycline	54671203	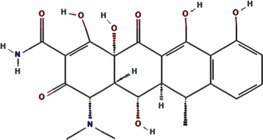	SARS-CoV-2 E (Cao et al., 2021)
Flavonoids	Rutin	5280805	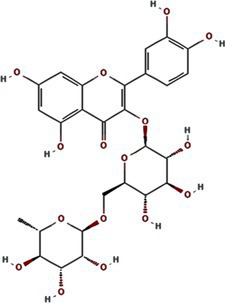	SARS-CoV-2 E (Cao et al., 2021)
	Kaempferol	5280863	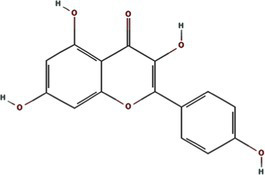	SARS-CoV-1 3a (Periferakis et al., 2023) SARS-CoV-1 E (Periferakis et al., 2023)
	Tiliroside	5320686	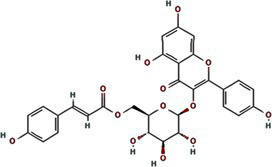	SARS-CoV-1 3a (Fuzimoto and Isidoro, 2020)
	Apigenin	5280443	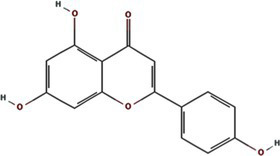	SARS-CoV-1 E (Fuzimoto and Isidoro, 2020)
	Quercetin	5280343	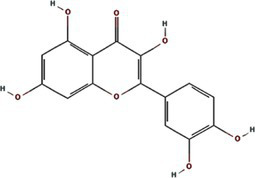	SARS-CoV-1 E (Gasmi et al., 2022) SARS-CoV 2 3a (Fam et al., 2023)
	Afzelin	5316673	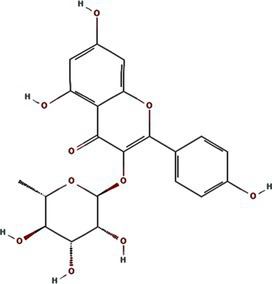	SARS-CoV-1 3a (Fuzimoto and Isidoro, 2020)
	Genistein	5280961	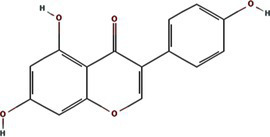	SARS-CoV-1 E (Breitinger et al., 2021) HIV-1 VPu (Awaluddin et al., 2023)
Anthraquinones	Emodin	3220	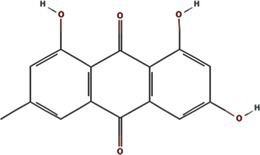	Human coronavirus 3a (Schwarz et al., 2011) SARS-CoV-1 3a (Schwarz et al., 2011) SARS-CoV-2 E (Toft-Bertelsen et al., 2021) SARS-CoV 2 3a (Yu et al., 2021) SARS-CoV-2 ORF7b (Toft-Bertelsen et al., 2021)
Vitamins	Tretinoin	444795	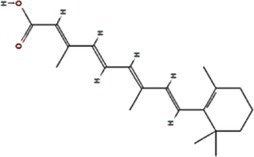	SARS-CoV-2 E (Dey et al., 2020)
*Bacillus* spp. metabolites	Cyclo (L-Leu-L-Pro)	7074739	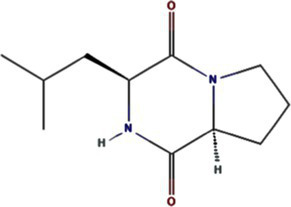	PVY 6K1 (Shen and Li, 2020)
	2-(3-Indolyl) ethanol	10685	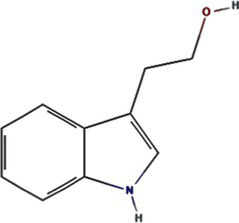	PVY 6K1 (Shen and Li, 2020)
	N-[2-(1H-indol-3-yl) ethyl]	70547	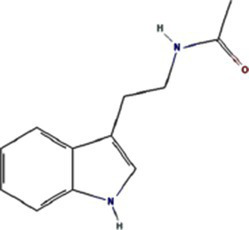	PVY 6K1 (Shen and Li, 2020)
	p-hydroxyphenethyl alcohol	10393	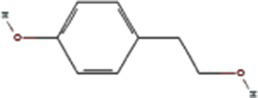	PVY 6K1 (Shen and Li, 2020)
Other compounds	Rhamnopyranosy	–	–	SARS-CoV-1 3a (Schwarz et al., 2014)
	EGCG	65064	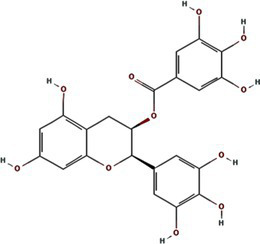	SARS-CoV-1 E (Breitinger et al., 2021) SARS-CoV 2 3a (Fam et al., 2023)
	Resveratrol	445154	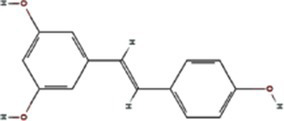	SARS-CoV-1 E (Breitinger et al., 2021) SARS-CoV 2 3a (Fam et al., 2023)
	Curcumin	969516	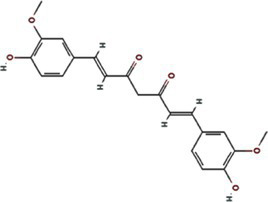	SARS-CoV 2 3a (Fam et al., 2023)
	BE-12	146403484	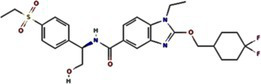	SARS-CoV-2 E (Xia et al., 2021)
	Xanthene	7107	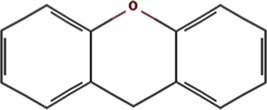	SARS-CoV-2 E (Xia et al., 2021) SARS-CoV 2 3a (Toft-Bertelsen et al., 2021)
	Capreomycin	3000502	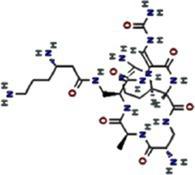	SARS-CoV 2 3a (Tomar et al., 2021)
	Darapladib	9939609	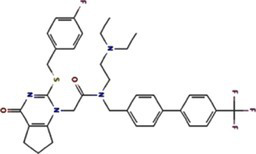	SARS-CoV 2 3a (Tomar et al., 2021)
	Verapamil	2520	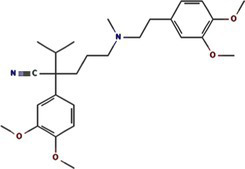	Classical swine fever virus P7 (Gladue et al., 2012)
	Gliclazide	3475	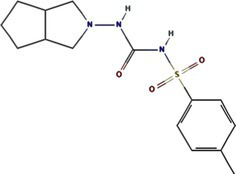	SARS-CoV-2 E (Singh Tomar and Arkin, 2020)

### 6.2 Biocontrol agents

Several species of the genus *Bacillus*, such as *Bacillus subtilis* and *B. licheniformis*, have demonstrated efficacy in controlling potyviral diseases (Miljaković et al., 2020; Xuan et al., 2024). These bacteria can inhibit the accumulation and spread of viruses through multiple mechanisms, including activation of the plant defense systems, secretion of antimicrobial compounds, and nutrient competition with other pathogens (Veselova et al., 2022; Amin et al., 2023; Xuan et al., 2024). Recent studies reveal that some *Bacillus* species secret compounds directly target potyviruses. For instance, nine organic compounds extracted from *B. pumilus* E303035 fermentation showed strong inhibition against PVY, among which cyclo (L-Leu-L-Pro), 2-(3-Indolyl) ethanol, N-[2-(1H-indol-3-yl) ethyl] and phydroxyphenethyl alcohol significantly inhibit the activity of PVY 6K1 (Shen and Li, 2020). Furthermore, these compounds also affect the expression of viral genes involving in viral proliferation (Shen and Li, 2020). In addition, *Bacillus* can also produce other metabolites, such as surfactin, iturin, foenomycin, hydrolases, and volatile organic compounds (Wang et al., 2018; Rajaofera et al., 2019; Jin et al., 2020). These compounds exhibit antiviral activities against various plant viruses, such as cucumber mosaic virus (CMV; *Cucumovirus CMV*), tomato spotted wilt virus (TSWV; *Orthotospovirus tomatomaculae*), pepper mild mottle virus (PMMoV; *Tobamovirus capsici*), and tomato yellow leaf curl virus (TYLCV; *Begomovirus coheni*) (Ongena and Jacques, 2008; Kong et al., 2018). In the future, it is necessary to identify the precise target of these active compounds and structurally optimize their structures for enhanced specificity. In conclusion, the secondary metabolites produced valuable resources for identifying potential 6K1 inhibitor.

## 7 Unresolved questions and future directions

Despite significant advances concerning the critical functions of 6K1 including increasing cell membrane permeability, facilitating viral replication, and interacting with host factors, several key knowledge gaps remained. For instance, the precise atomic structure of 6K1, its ion selectivity of 6K1, and its interaction network with viral and host proteins. In addition, investigation of the evolutionary pressures shaping 6K1 diversity across potyviruses and potential role of 6K1 in virus-vector interaction will underscore its functional diversity. Finally, screen small molecule antiviral agents that targets 6K1 will significantly benefit the control of potyviral diseases. In conclusion, 6K1 is a multifunctional protein represents the central hub in potyviral infection, bridging replication, membrane dynamics, and host manipulation and the discovery of 6K1 as a plant viroporin not only enhances our understanding of the pathogenesis of potyvirids but also provides an important target for developing antiviral agents.
