# Supplementary figures and images for: The 6-kilodalton peptide 1 of the family Potyviridae: small in size but powerful in function
Source: Front Microbiol. 2025 Jun 5;16:1605199. doi: 10.3389/fmicb.2025.1605199 (PMC12176749; doi:10.3389/fmicb.2025.1605199)

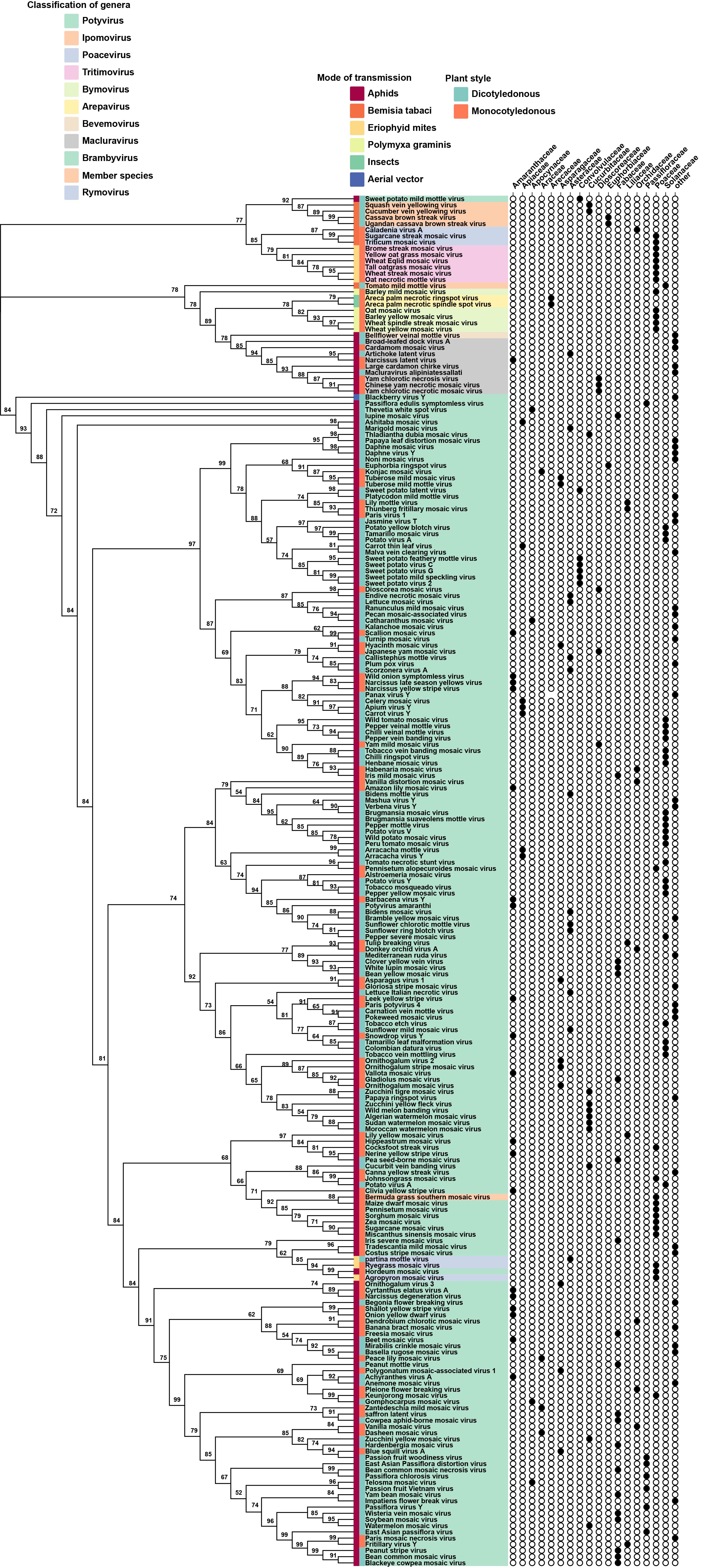

Supplement: Supplementary file 1 [file Image_1.jpg]
