# Supplementary material for: The 6-kilodalton peptide 1 of the family Potyviridae: small in size but powerful in function
Source: Front Microbiol. 2025 Jun 5;16:1605199. doi: 10.3389/fmicb.2025.1605199 (PMC12176749; doi:10.3389/fmicb.2025.1605199)
Supplement: Supplementary file 2 [file Image_2.pdf]

```
                                       1         10             20             30             40
22.Asparagus        ..........HKSKSQDTLQKIVAFVALI.MMTFDADRSDCVYKILNKLKGIIGTIE...
169.Ashitaba        .........AKKSKGEATLERIVAFMSLI.MMMFDADKSDYIYKVLNKLRSLTTTIETEE
216.Marigold        .........ASKRKSEADLERIVAFVSLI.MMLFDSEKSDCVFRVLNKLKSLVGTIDNE.
8.Agropyron         .........AKDTNHIW.TEKCIATFVLV.MMMFDTDRSDKLYSTLNKLKGVFSTIGQD.
59.Hordeum          .........AKDNNHIW.TEKCIATFVLL.MMMFDVEKSDKLYSTLNKLKGIFSTIGQG.
9.Ryegrass          .........AKDDTNVW.IEKCIATIVLM.MMAIDSNKSDKLYQILCKLRTVFSTMGQT.
7.partina           .........AKTKQQVN.YEKIIALMTLF.MMYFDNVKSDVLYSTLNKLKTIFSTLGQE.
121.Tradescantia    .........AYKTGTKK.IEQSIAFITLI.MMLIDVDKSDALYHILNKIKGIFSTLPEK.
180.Costus          THTEVQQQYSNDTTTKK.IEQIIAFSTLL.LMLFDQERSDALFRTLMKCKGVFSGLGQ..
63.Iris             .........AGTPSTRK.LESAIAATALV.MMIFDSNRCDVLVKILNKLKTIFAALNFG.
26.Bean             .........AKNATQLQ.FEKIVAFMALL.TMVIDTERSDAIFKILSKLKTVFQTMGEN.
31.Blackeye         .........AKNATQLQ.FEKIVAFMALL.TMLIDTERSDAIFKILSKLKTVFQTMGET.
12.Peanut           .........AKNANQLQ.FEKIVAFMALL.TMVIDTERSDAIFKILSKLKTVFNTMGDN.
49.East             ................Q.FEKIVAFMALL.TMCIDTERSDAIFKILNKLKVVFSTMGED.
53.Fritillary       .........SKEAIQLQ.FEKIIAFMALL.TMCIDTERSDAIFKILSKLKTVFNTMGED.
204.Paris           .........GKTAVQLQ.FEKIVAFMALL.TMCIDAERSDAIFKILSKLKTVFTTMGED.
61.Impatiens        .........AKTGTQVQ.FEKIIAFMALI.TMIIDTERSDAIFKVLNKLKSVFQTMGDN.
134.Yam             .........AKTATQVQ.FEKIIAFMALL.TMVIDTERSDAIFKVLGKLKTVFSTMGEE.
207.Passiflora      .........GKTAIQSQ.LEKIVAVMALI.TLVIDGERSDAIFKTLSKIKTFFSTMGED.
107.Soybean         .........AKTATQLQ.LEKIVAFMALL.TMCIDNERSDAVFKVLSKLKAFFSTMGED.
130.Watermelon      .........AKTATQLQ.LEKIVAFMALL.TMCIDNERSDAVFKILSKLKTFFGTMGEE.
133.Wisteria        .........AKTKLQLQ.LEKIVAFMALL.TMCIDNERSDAIFKILSKLKTIFGTMGED.
25.Bean             .........AKTQTQLQ.LEKIVAFMALL.TMCIDSERSDAVFKILQKLKSAFGTMGED.
85.Passiflora       .........SKSHTQLK.LEKIVAFMALL.TMCIDSERSDAVFKILRNIKTVFGTMGED.
205.East            .........SKTVNQLQ.LEKIIALMALI.TMCIDHDRSDAVFRILHKIKTVFGTIGEE.
181.saffron         .........AKTEKQRQ.LEKIVAFMALL.AMVIDSERSDAVSKVLSKLKSVFITMGEE.
45.Cowpea           .........AKTHSQVH.LEKIVAFMALL.TMCVDAERSDAIFKILNKLKSVFGTMAEE.
223.Cowpea          .........AKTHSQVH.LEKIVAFMALL.TMCVDAERSDAIFKILNKLKSVFGTMAEE.
116.Telosma         .........GKTGTQVQQFEKIIAFMALL.TMVVDSERSDALFKILNKLKTVFGTMGEE.
206.Passion         .........SKSATQVN.FEKIIAFMALL.TMCVDAERSDALFRILHKLKTVFGTMGED.
136.Zantedeschia    .........AKTAAQLQ.LEKVVAFFAIL.TMCFDTERSDAVFKVLNKLRAVFLTLGEG.
13.Vanilla          .........AKTALQLQ.FEKIIAFLAIV.TMCIDSERSDAVFKLLSKLKTVFATVGEN.
128.Vanilla         .........AKTALQLQ.FEKIIAFLAIV.TMCIDSERSDAVFKLLSKLKTVFATVGEN.
48.Dasheen          ...............LQ.FEKIIAFLAIV.TMCVDAERSDAVFRLLSKLKTVFATV....
86.Passion          .........AKTQVQLQ.FEKIIAVLALI.TMCIDAERSDAIFRILSKLKMVFSTVGED.
177.Blue            .........AKTAVQLQ.FEKIIAVLALL.TMCIDAERSDAIFRILSKIKTVFTTMGED.
56.Hardenbergia     .........AKTAVQLQ.FEKIIAILALA.TMCIDAERSDAVFRILSKIKMAFSTVGED.
138.Zucchini        .........AKSAVQVQ.FEKIIXXLXLL.TMCFDAERSDAXFKILTXXKTVFSTVGGT.
74.Narcissus        .........YKKKNEND.LEKIVATIALI.MMIFDTDRSDAVFKILNKVKTVFSTFGER.
184.Cyrtanthus      .........HKRKSEND.LERIVAVIAMI.MMVFDSSRSDAVFKILNKLKAVFGTFNER.
81.Ornithogalum     ........AKRTANEVY.LEKIVAMIALS.TMVFGSECSDGVFKILNKLKTVFTTMHTS.
18.Anemone          .........AKTKTEHN.LEKVMAFMALI.TMMYDTEKSDAVFRVLGKVKTIFGTLGEE.
213.Polygonatum     .........AKTASELN.FEKIIAFMALI.TMIADTERSDAVFRTLGKVKTIFG.....
166.Achyranthes     .........SKTKSELH.LEKVVATMALF.TMILDPEKSDAVFRVLSKVKTFFGTVEDT.
89.Peanut           .........AKTETELG.LEKVVAYLALI.AMIFDGERSDAVFRALSKLKTVFGTLGET.
24.Basella          .........SKNPFERN.LEKVVAMMALF.AMVFGSDKSSAVFNVLRNIKTVFGTLEDG.
88.Peace            .........SKNPFERN.LEKVVAMMALF.AMMFGSDKSSAVFNVLRNIKTVFGTLEDD.
198.Mirabilis       .........AKNRCERS.LEQVIAMMALM.AMLFGSDKSSAVFNSLRNIKTVFSTIEDE.
183.Keunjorong      .........SKTYLEKN.FEKIIAFMALI.TMMFDDKKSDAVFKCLGKIKTVFGTMEDV.
191.Gomphocarpus    .........NKTYIERN.FEKIIAFMALL.TMMFDEKKSDAVFKCLGKIKTVFGTMDDV.
212.Pleione         ..........KAYLERN.FEKVIAFMALI.TMIFDERKSDAVFKCLGKIKTVFNTMSDV.
28.Beet             .........ANKVYEKK.LEKAVALMALF.TMIFDTEKSGAVFSILRNIKSVFSTLGEE.
52.Freesia          .........GKSPTEKS.LERTVAMVALL.AMVFDTERSDAVFKILSKIKSVFSTLGDE.
23.Banana           .........SKTVFESG.MERVVAVFALL.AMIFDTSKSDAVFRILQKFKTCIASINNR.
185.Dendrobium      .........AKSPYEQG.LEKTVGIFALV.AMIFDTARSDAVFRILSKLKTVFSLIDDK.
78.Onion            .........AKSKSETT.LEQIVALCALM.AMFFNTEKSDAVFKILSKIKNVFSSTDFP.
104.Shallot         .........AKSQSEVT.LEKIVAYVALF.AMLFNSEKSDGVFKILSKLKTIFSTTD...
29.Begonia          .........NKSESEMQ.LEKIVAFVALI.AMMYNTEKSDAVFRILNKLK..........
77.Nerine           ..............ERK.LEQIIAIMALA.AMMFSPGRSDCVFKLLSKVKTIFSTVE...
87.Pea              .........AKKHGEIR.FEQTVALMALL.AMMFGSDRSDAVFSTLSKVRTIFTTMAQE.
43.Cocksfoot        .........AKTHAEAK.LEQIMARMALA.AMMFDAQRSDAVFKVLSKIKTVLTSAGQS.
58.Hippeastrum      .........SKSKQEHI.FEQIIGFLLLA.TLMFSPARSDVVFKVLNKVKSVLTSTAPD.
65.Johnsongrass     ..........KGYAELK.LEQVIAFATLL.TMLYDAERSDAVYKILQKIKACTNICTQE.
97.Pleioblastus     .............SEIR.LEQVIAFATLA.TMLYDADRSDAVFKILQKI...........
34.Canna            .........KKGSTELQ.LEQAIAFATLL.TMLFDADRSDAVFRILQKIRSCTQIIGTT.
182.Cucurbit        ..........KGPTEQN.LEKVIAFMVLL.AMFVDANRSDAIASVLNKVKNITTTLSTP.
71.Maize            .........GKSNLEIN.LEQAMAIGTLI.TMIFDPTKSDAVYKVLNKMRTILSTVEQD.
165.Bermuda         ..................LEQAMAIGTLI.TMMFDPTKSDAVYKVLNKMRTILSTIEQ..
199.Miscanthus      .........GKSHLETN.LEQAMALGTLI.TMIFDPVKSDAVYKVLNKMRTVINTFEQN.
225.Zea             ..........KSRLETN.LEQAMAFGTLL.TMIFDPNKSDAVFKVLNKMKTVINTFEQ..
108.Sugarcane       .........GKSNLETN.LEQAMAVGTLL.TMILDPQKSDAVYKVLNKMRTVISTFEQN.
90.Pennisetum       .........HKSKLETN.LEQAMAVGTLL.TMMFDPNRSDAVYKVLNKMKTVINTYEQN.
208.Pennisetum      ..................LEQAMAVGTLL.TMMFDPNRSDAVYKVLNKMKTVINTYEQ..
106.Sorghum         ..........KSNFETN.LEQAMAVGTLL.TMVLDPVKSDAVFKVLNKIKTCINTYEQN.
178.Clivia          .........SKGLCEVR.LEQSIAIVTLL.AMIFDQERSDAIYKILNKIRTLIMTTEQN.
35.Carnation        ..........AKPTEIVQMEKIIAVTALI.MMIFNAERSDCVHRVLQKFSSVMTTLHAP.
195.Lettuce         ..........SK.KEIKILEKCIAFTTLI.LMMFDSERSDGVYKILSKFKGVMGTIDSQ.
44.Colombian        .................YMERTIAVITLL.VMAFDAERSDCVYKILSKFKGLVVSVD...
172.Tamarillo       ..........KGKKEFAYMERIIAIITLL.IMAFDAERSDGVYQILNKFKGVIGSAERET
105.Snowdrop        ..........AKTKRFIVMERTLAYITLI.VSLFDMERGDGVFQILNKFKSLFVSIEDP.
118.Tobacco         ..........AKQPEIAYFEKIIAFITLV.LMAFDTERSDGVFKILNKFKGILSSTEREI
193.Sunflower       ...........SKTDMVRLEKIIAYATLI.MMAFDTERSDGLYKILNKFKGIISSTNKES
68.Leek             ..........AKGD.FQHLEKMIAMLVLL.TMLFDANRSDAVYKILNKFKGVMSSIDKEP
98.Pokeweed         ........QDAKSD.SRKMEQGIAMIALI.LMVFDTDRSDCVYRTLNKFKGVMSSLYAKP
217.Paris           ..........AKSHSIQALEKVIAFIALI.LMTFDAERSDGVARILGKFKQVLEPLQREP
224.Lily            ..........HKGKSLVNLERIIALFTLA.LMIVDPDRSDGVYKLLTKLKSVITTATQEP
27.Bean             .........AKPNDMIA.LEKIVAVTALI.LMIFDAERSDCVYKVLNKLKGILSTTTQDA
210.White           .........AKPNDMVA.LEKVVAVTALI.LMIFDAERSDCVYKVLNKLKGILSTTTQ..
42.Clover           ................A.LEKVVAFTALV.MMIFDGERSDCVYKILNKLKGIISTTTQ..
214.Mediterranean   .........AKSNEMIQ.LEKVIATVALI.MMVFDQERSDCVYKILNKLKGVINTMNQDN
117.Thunberg        .........AKTDGERN.LERVVAVSALL.LMIFDAERSDVVYKILNKLKTLIGVVETEP
203.Paris           .........AKTDGERN.LERVVAVSALL.LMVFDAERSDVVYKVLNKLKNLIGVVEAEP
70.Lily             .........AKPDGERN.LERIMAIIALT.MMVFDADRSDCVYKVLNKLKGLLNTVHQEP
111.Sweet           .........AKSPTEAK.YERIIAMIALF.MMAFDAERSDCVYKILNKLR..........
211.Platycodon      .........AKSQEQAQ.LERIIAVIALI.LMVFDAERSDCVYKILNKLRTLVGISSSDT
124.Tulip           .........AKSNDAQK.LEQVVAFISLI.LMLFDAERSDCVYKILNKLKGLMSTI....
167.Donkey          .........AKSKDMFK.LEQCVAFVALV.LMLFDNERSDAVFKILNKLKTTFTTICGGV
73.Moroccan         ..........AKTVNEKRMEQILAFVTLI.MMFIDAEKSDCVYRVLNKFKGVVGTIEQD.
179.Sudan           ..........AKTQNEKRMEQILAFVTLI.MMFIDAEKSDCVYRLLNKFKGVIGTIEQD.
14.Algerian         ..........AKTVNEKNMERILAFLTLI.MMMVDADKSDCVYKLLNKFKSVVGTIEQD.
197.wild            ..........AKTAEERRLEQIIAFFTLL.MMMVDAERSDCLYRILNKFKGVVGTIEQD.
137.Zucchini        ..........AKTDNEKTMEQIVAFMTLL.MMLFDTERSDCLFRVLNKFKGVVGSIGQN.
84.Papaya           ..........AKSDNEKKLEQVIAFITLI.LMMIDVDKSDCVYRILNKFKGVIKSCNSD.
```

```
209.Zucchini         ..........AKTNNEKKLEQIIAFITLI.MMMVDTDKSDCLYRILNKFKGIMASDATN.
79.Ornithogalum      ..........AKNPQEHQLEQVIAFMTLL.SMMYSPERSDGLFKILNKVKGVLGTIEGG.
190.Gladiolus        ..........AKNPQEHQLEQVIAFMTLL.SMMYSPERSDGLFKILNKVKGVLGTIEGG.
202.Ornithogalum     ..........VKNQQEQNLEKIIAFVTLL.SMLYSPERSDSLFKILNKVKGVLGTIEGG.
126.Vallota          ..........AKNAKECQLEQIIAFMTLL.SMLYSPERSDCLFKALNKVKGVLGTIEND.
80.Ornithogalum      ..........AKNVEERKLEQVIAVMTLI.AMLYSPERSDCLYKILNKVKGILGTVESD.
95.Pepper            ......................VAFISLV.IMMFDAERSDCVFKTLNKFKGIISSLDSE.
201.Tobacco          ..........RGTADIKKLEHIXAFISLV.IMVFDADRSDCVFKTLNKFKGIISSMDSE.
101.Potato           ..........RSTPGVKNLEQVVAFMALV.IMVFDAERSDCVFKTLNKFKGVLSSLDHE.
15.Alstroemeria      ..................LEHVVAFITLV.AMVFDAERSDCVFKILNKLKGIVSSLDSE.
32.Bramble           ................KNLEQVVAFITLV.MMVFDSERSDCVFKTLNKLKGVVSTLDYG.
173.Bidens           ..........QSTTGVKNLEQVVAFITLV.MMVFDSERSDCVFKTLNKLKGVVSTLDYG.
92.Pepper            ..........KATAGVKNLEQIIAFITLV.TMLFDSERSDCVFKTLNKLKGTVSTLDTN.
109.Sunflower        ..........KSTPEVKNLEQVIAFITLI.TMLFDSERSDCVFKTLNKLKGIVSTLDCE.
192.Sunflower        ..........KSTAGVKNLEQIVAFVTLI.TMIFDSERSDCVFKTLNKLKGVVSTLDME.
16.Potyvirus         ..........KATPEVKNLEQIVAFITLV.IMMFDPERSDCVFKTLNKLKGAVGTLDYD.
171.Barbacena        ..........RHTSEVKGLEQVVAFITLI.FMTFDSERSDCVFRTLNKLKSVVGSLDND.
188.Tomato           ..........KVTEGTKKIEQIVAFMTLV.MMMFDAERSDCVFKTLNKLKSTISTMDYE.
189.Pennisetum       ..........KVTEGTKKIEQIVAFMTLV.MMMFDAERSDCVFKTLNKLKSTISTMDYE.
20.Arracacha         ................QKLEQIIAFVVLV.FMMFDAERSDCIFKTLNKLKGVVATMDRQ.
21.Arracacha         ................QKLEQIIAFVVLV.FMMFDAERSDCIFKTLNKLKGVVTTMDRQ.
129.Verbena          ..........KNDQDLKKLEHIVAFITLV.MMVFDSERSDCVFKTLNKFKGVVASLNST.
222.Verbena          ..........KNDQDLKKLEHIVAFITLV.MMVFDSERSDCVFKTLNKFKGVVASLNST.
174.Brugmansia       ..........KSFDELKKLEHIVAFISLV.VMVFDAERSDCVFKTLNKFRGIVGSLNSN.
220.Mashua           ..........KSESDLKSLEQIVAFVTLI.IMVFDAERSDCVFKALNKFKGVVSSLNSN.
30.Bidens            ................RQIEQIIAFVTLV.LMTFDAERSDCVFKTLNKFKGTVSSLNST.
17.Amazon            ..........KATSEMKHLEQIVAFVALV.LMVFDSEKSDCVFKTLNKFKGIIGSLNST.
96.Peru              ..........DSKSDLKKIEHIIAFVSLA.VMLFDVERSDCVFKSLNKFKGVISSLNSD.
131.Wild             ..........DSKSDLKKIEHIIAFVSLA.VMLFDVERSDCVFKSLNKFKGVIGSLNSD.
100.Potato           ..........ASKSDLKKIEHIIAFVSLA.VMLFDVERSDCVFKSLNKFKGVIGSLNSE.
91.Pepper            ..........RSTEDLKKVEHIIAFVTLA.IMLFDSERSDCVFKTLNKFKGVVCSLGSG.
33.Brugmansia        ..........RSTPDLKKLEHIIAFTSLL.IMLFDTERSDCVFKSLNKFKGLVSSMDSD.
54.Gloriosa          .................RIEQILAFVVLI.MMLIDAERSDCVYKILNKFKGVIRSIEP..
120.Tobacco          ..........AKNTGQASLERIIAFVSLT.LMLFDNERSDCVYKILTKFKGILGSVENN.
50.Endive            ..........ARSQGQTELERIVAFAALV.MMMFDSERSDCVYKVLNKLKGCISSIEGG.
110.Sweet            .........GKSRKEMQ.YERIIAFVSLL.LMIVDSEKSDCVYKILQKLKGLMGTINND.
114.Sweet            .........GKSRKEIQ.YERIIAFISLL.LMVIDSERSDCVYKILSKLRGLMGCIDGG.
113.Sweet            .........AKSAKESS.YERIIAFIALV.LMVIDAERSDCVYKSLNKLKGLMGTIGDG.
187.Sweet            .........AKSAKESS.YERIIAFIALV.LMVIDAERSDCVYKSLNKLKGLMGTIGDG.
115.Sweet            .........AKGLKEAN.YERIIAFIALI.LMVVDAERSDCVYKALNKLKGLMSTICGGP
36.Carrot            .........KKRIEEIQ.MERIIAFVSLV.MMMFDAERSDCVYKILNKLKGLTNTIA...
99.Potato            .........AKASEQVN.FERIIAFISLVILMMFDCERSDCVYRSLTKLKSLMSTVENT.
170.Tamarillo        .........AKASEQVN.FERIIAFVSLV.LMMFDSERSDCVYRSLTKLKSLMSTVENT.
221.Potato           .........SKSSEQVN.FERIIAFVSLV.LMMFDAERSDCVYRSLTKLKSLMGTVENT.
194.Jasmine          .........AKNAEQIK.FERIVAFISLV.LMAFDSERSDCVYKILMKLKNLIGTCEQD.
72.Malva             ...................ERIIAFISLI.LMMFDAEKSDCVYKILSKLKGLINTTEQG.
103.Scallion         .........SKRQEQQE.LERIIAFVALV.LMMFDAERSDCVTKILNKVRNLVTTTEST.
125.Turnip           .........AKRQSEQE.LERIIAFVALV.LMMFDAERSDCVTKILNKLKGLVATVEPT.
66.Kalanchoe         .........AKRQSEQA.LEQIIAFVALL.LMMVDPERSDCVTKILTKLKNLVTTIDQD.
11.Plum              .........SKRDSQAN.LERVVAFVALV.MMLFDSERSDGVYKILNKLKSVMGSIDQT.
215.Scorzonera       .........AKRKSEAD.LERVIAFVALV.MMLFDAERSDGVYRTLCKLKGLMGCVDQ..
127.Vanilla          .........VKQHSCQQ.LEKVVAFVALV.MMMFDAERSDCVYRTLNKFKGIMGSIDSG.
60.Hyacinth          .........AKKECEVE.LERVIAFMALV.MMVFDNERSDYVHRALSKVKSLMSSIDSD.
19.Apium             .........AKRVSEAR.LEQAMAFVALI.LMAIDSERSDCVHKVLNKLKSLMSIADAD.
37.Carrot            .........AKRVSEAR.LEQAMAFVALI.LMAIDAERSDCVYKVLNKLKNLMSIADAD.
39.Celery            .........AKRVTEAR.LEQAMAFVALV.LMAIDSDRSDCVYKVLNKLKSLMTIADAD.
82.Panax             .........AKRNSEVR.LEQAMAFVALI.LMIFDSEKSDCVYKVLSKLKNLMSIADTS.
75.Narcissus         .........VKRRSEQE.LEKVVAFIALI.LMMFDSERSDCVAKILQKLKNLISSAEPD.
76.Narcissus         .........TKRKSEQE.LERVVAFVALV.LMMFDCERSDCVTKVLTKLKNLMSSVEPN.
168.Wild             .........ARRKSEQD.LEKIVAFVALI.LMMFDSERSDCVAKVLNKLKNIMSSADPT.
64.Japanese          .........AKRRGELE.LERVVAFIALV.MMVFDSERSDCVVKILNKLKNIISSTDAD.
175.Callistephus     .........AKRESEAK.LEQVVAFTALL.LMLVDSERSDGVYKILSKLKGIMSSVDGP.
40.Chilli            .........AKRESEVR.LEQIVAFMALV.MMIFDNDRSDCVYRVLNKFKNIVSTAEQD.
119.Tobacco          ........AVKRASEVR.LEQIVAFIALV.MMIFDNDRSDCVYRVLNKFKNIVGVADQE.
55.Habenaria         .........NKRESETR.LEQIVAFIALV.LMVFDNERSDCVYRVMNKLKNVMSVAEQD.
62.Iris              ........ATKRESEVR.LEQIVAFTALV.LMVFDNERSDCVYRVMNKFKNIVSVADQD.
57.Henbane           ........AVKRESEAR.LEQVVAIIALL.MMVFDNERSDCVYRVMNKLK..........
93.Pepper            ........ASKRPSEAK.LEQIVAFVALM.MMVFDTDRSDCVYKVLNKLRNVMGVVDNDA
94.Pepper            ........ASKRPAEAK.LEQIVAFVALM.MMVFDTDRSDCVYKVLNKLKNVMGVVDNDA
41.Chilli            ........ASKRPSEAK.LEQIVAFIALM.MMVFDTDRSDCVYKVLNKLRNVMGVVD...
132.Wild             ........ASKRPSEAK.LEQIVAFIALL.MMVFDGDRGDCVYKVLNKLRNVMGSVDNEA
83.Papaya            .........AKRKGEQN.LEKIIAFVALV.MMIFDSEKSDCVYKTLNKLRNLVATCDEP.
219.Thladiantha      .........AKRNSEQN.LEKIIAFVALV.LMIFDSEKSDCVYKTLNKLRNLVSTCDEP.
135.Yam              .........AKRESEQR.LEQIIAFIALV.MMVFDNERSDCVYKVLNKLKNLMNTA.EP.
122.Tuberose         .........AKPPTNVQ.FERIIAIVSLF.LMVFDSARSDAVYRSLQKLKTLTGIANGP.
123.Tuberose         .........AKAAPNAN.FERIIAIVSLI.LMVFDCSRSDAVCRSLQKLKFLTSTATDT.
67.Konjac            ..........RSANEAA.FESIIAITSLV.LMVFDQERSDCVYRILQKLRSLVGISGEI.
51.Euphorbia         .........ARRPSQAN.VEKVIAFVSLF.LMLFDSERSDCVYKSLLKVRTLFNIAENE.
200.Noni             .........ASRGNNAK.YEQLIAFVALI.LMVFDADKSDCVYRILNKLKSLTGTLENS.
38.Catharanthus      .........SKGENTNN.LEKYMAVAALV.ALVFDSARGDAVYRILTKIKTLTSVASQD.
176.pecan            .........SKGRGQNK.LEQIIAMAALV.MMMFDSERSDAVYRSLQKLKFLTTTADDC.
69.Lettuce           ...............AN.LERIIAFTALV.MMMFDSERSDCVYRSLSKLKSLVSTCEDD.
102.Ranunculus       .........SKKAEQAK.LEQIIAFVALV.LMMFDSERSDCVYRALTKLKQLVSITNTP.
186.Dioscorea        ........ASNRTEQQR.LEQIVAFVALV.MMMFDANRSDCVYRILNKLKTLVSGAEGL.
196.lupine           .........SKVSAGEAKLEKTIAVAALI.MMIFDSERSDYLYKTLNKLKSLVRTVDDD.
46.Daphne            .........SKSAESQQ.YEKSLAFVALL.LMVFDSERSDCVYKVLCKLRALTNICGER.
47.Daphne            .........AKNVENQK.FEKIIAFIALV.MMIFDTQRSDCVYKALTKIRSLTTICGET.
218.Thevetia         ..........AKHSSEK.IEKVVAFSALV.MMLFDSDRSDYLYKLMNKLRGLVTTCER..
161.Blackberry       ..........SADEGKN.MCKIFASAILV.MMVFDAHRADLMYKSFSQVRALFNTLYDSG
10.Passiflora        .........SSKRTSGQVPAKFMALAAIV.TALFNNDLSDQWYSCMVKFKSLMSTMFDEY
2.Wheat              ..........GK.REEAWITRVMAVLYI.IASLFSVDFSSALYSNLVKFRTIFDILKFN.
3.Oat                ..........SK.KEEAWITRTMAVLYI.ISSLFSVEFSSALYSNLVKFRTIFDILKVN.
4.Tall               ..........GK.REEAWISKVMAMMYI.VATFFSVDFSSAIYSNLVKFRTIFDILKVN.
6.Yellow             ..........GK.AEEAWITKALALMYL.VSTLFSMDLSSALYSNLVKFRTIFDILRAN.
5.Wheat              ..........ARGKEDMWITRFMASCYI.VSALFSWDISDSIYSSMVKFRTIFDILRVN.
1.brome              ..........SK.QEELFISKFMAVCFV.ISTFFSLDMSNAIYSSLTKFRAIFSILSVG.
140.Sugarcane        ..........QG.KADNMYVKILAWISL.LVGCFNVGLANDIYFAVTKYRTLLDIATTSS
141.Triticum         ..........KA.ASDSLYIQCLAWLAL.VVGCFNSGLANDIYFSTTKYRTLLDMVKTAH
139.Caladenia        ..........GK.QEADFYIKCMAWLAL.IASVFNLNFGDQIYKNTTKFRTLLAIVTAPS
151.Cassava          ..........GR.REESFVLRWCAFATL.FLSFINYDWAVGSVSAIGKMKTVFGALGPD.
155.Ugandan          ..........GR.KEESFVLKWCAAATL.FLSFLNYDWAVGCVSAIGKMKTIFSALGPD.
152.Cucumber         ..........GK.KEEATIMKWCAMLTL.VMSFINFDWALASVSALGKMKTIFSALGPN.
153.Squash           ..........GK.KEEAFVLKWCAFLTL.LMSFFNFDWALASTTAIGKLKTLYGVLGSE.
```

```
112.Sweet          .........HGK.KEEAFLFKICAIFAL.IAGIVDYEWGAAACATMNKVRSICTVLGSV.
142.Artichoke      ...............EKQMMAAMAMMTL.VVHAFDIDLAITMSGALNHVARLANMLTDT.
148.Narcissus      .........GPHKSSEKQMMAAMAMITL.IVHAFDMDLAITMSSAQNHVGRLANMLTDT.
145.Chinese        .........AYGKSSEKQMMAAMAMVTL.FVHAFDMDLALMMSNSLNHVARLANMLTDT.
150.Yam            .........AYGKSSEKQMMAAMAMITL.FVHAFDMDLALMMSNSLNHVARLANMLTDT.
149.Yam            ............KSSEKKMMSAMAMITL.FVHAFDMDLALMMSNSLNHVARLANMLTDT.
146.Large          ...............EKQMMSVMAMITL.LVHAFDIDLAIMMSNSLNHVARMANMLTDT.
147.Macluravirus   ...............EKQMMSAMAMLTL.LVHAFDMDLAVMMSNSLNHVARLANMLTDT.
144.Cardamom       .........ASIKSSEKQMMSVMAMITL.LVHAFDIDLAITMSNSLNHVARMANMLTDT.
143.Broad-leafed   .........LNSKENEKKMMAAMAMITL.FVHAIDMDLALVMTSSLNHVARLVGLLTDS.
162.Bellflower     ............TTNEKKVTGLIAFIVL.AVHIFDADLAIMLSSSLHTISRTASMLTDE.
159.Wheat          ........ARSANEKEKKLMMLLASAVG.ITYLFDYDIAEALGNCLHKVSRLSSYLIDD.
160.Wheat          ........ARSANEKEKKLMMLLASAVG.ITYLFDYDIAEALGNSLHKISRLSSYLMDD.
157.Barley         ........ARSTAEKEKKLMMILASVVG.ITYLFDYDIAEALGNCLHKISRLSSYLLDD.
158.Oat            ........ARNAPESEKKLMMLLASVVG.VTYLFDYDIAETMGNCLHKISRLANYLMDD.
163.Areca          ..........KKEEPPKVLMSFMAFAIL.ITYIFSVDTANMLSAAFNHFSRLAAALIDT.
164.Areca          ..........KKDEPPKMLMSFMAFAIL.ITYIFSVDTANMLSAAFNHFSRLASALIDT.
156.Barley         ...........ASQKDKRLIGILAFCIT.VIYMFDVDLADSLSNNLHKISRLVNLFLDD.
154.Tomato         ...........SKEKETWLMRWLAVFSMF.MYVIDVDWGNSLHSSVMKVKGLYALWQHDE
```

```
22.Asparagus         .........................................
169.Ashitaba         L...VRHQ.................................
216.Marigold         ....VYHQ.................................
8.Agropyron          ....AVYHQ................................
59.Hordeum           ....SVYHQ................................
9.Ryegrass           ....VVTHQ................................
7.partina            ....TIMLQ................................
121.Tradescantia     ....VEFQ.................................
180.Costus           .........................................
63.Iris              ....VRFE.................................
26.Bean              ....VQIQ.................................
31.Blackeye          ....VQIQ.................................
12.Peanut            ....VQMQ.................................
49.East              ....VKIQ.................................
53.Fritillary        ....VMIQ.................................
204.Paris            ....VKVQ.................................
61.Impatiens         ....VQTQ.................................
134.Yam              ....VRVQ.................................
207.Passiflora       ....VRVQ.................................
107.Soybean          ....VKVQ.................................
130.Watermelon       ....VKVQ.................................
133.Wisteria         ....VKAQ.................................
25.Bean              ....VRPQ.................................
85.Passiflora        ....VRVQ.................................
205.East             ....VRVQ.................................
181.saffron          ....VRVQ.................................
45.Cowpea           ....VRVQ.................................
223.Cowpea           ....VRVQ.................................
116.Telosma          ....VRMQ.................................
206.Passion          ....VRIQ.................................
136.Zantedeschia     ....VRVQ.................................
13.Vanilla           ....VRIQ.................................
128.Vanilla          ....VRIQ.................................
48.Dasheen           .........................................
86.Passion           ....VKVQ.................................
177.Blue             ....VKLQ.................................
56.Hardenbergia      ....VRLQ.................................
138.Zucchini         ....VRLQ.................................
74.Narcissus         ....VQFQ.................................
184.Cyrtanthus       ....VQFQ.................................
81.Ornithogalum      ....VEFQ.................................
18.Anemone          ....VRYQ.................................
213.Polygonatum      .........................................
166.Achyranthes      ....VRYQ.................................
89.Peanut            ....VRYQ.................................
24.Basella           ....VRYQ.................................
88.Peace             ....VRYQ.................................
198.Mirabilis        ....VRHQ.................................
183.Keunjorong       ....VRLQ.................................
191.Gomphocarpus     ....VRLQ.................................
212.Pleione          ....VRPQ.................................
28.Beet              ....VKYQ.................................
52.Freesia           ....VKYQ.................................
23.Banana            ....VGFQ.................................
185.Dendrobium       ....VHHQ.................................
78.Onion             ....VQYQ.................................
104.Shallot          ....VHYQ.................................
29.Begonia           .........................................
77.Nerine            .........................................
87.Pea               ....VRCQ.................................
43.Cocksfoot         ....VHHQ.................................
58.Hippeastrum       ....CRFQ.................................
65.Johnsongrass      ....VKHEG................................
97.Pleioblastus      .........................................
34.Canna             ....VEHQ.................................
182.Cucurbit         ....VHHQSEFYTKSGSLLTLENVYHQS.............
71.Maize             ....VPFPRIDFTNIFRTQVTHQ..................
165.Bermuda          .........................................
199.Miscanthus       ....STFPRLDFTSIFTTPVKHQ..................
225.Zea              .........................................
108.Sugarcane        ....VSFPSINFTNILTPPVTQQ..................
90.Pennisetum        ....ETFPRFDFTKMFNRNVVHQ..................
208.Pennisetum       .........................................
106.Sorghum          ....ATFPTVNFSSLLGTQ......................
178.Clivia           ....VRHE.................................
35.Carnation         ....VHFQ.................................
195.Lettuce          ....VHHQ.................................
44.Colombian         .........................................
172.Tamarillo        ....IRLE.................................
105.Snowdrop         ....VRFQ.................................
118.Tobacco          ....IYTQ.................................
193.Sunflower        ....IHLQ.................................
68.Leek              ....MTLQ.................................
98.Pokeweed          ....VSFQ.................................
217.Paris            ....MKFQ.................................
224.Lily             ....MRFQ.................................
27.Bean              ....YRFQ.................................
210.White            .........................................
42.Clover            .........................................
214.Mediterranean    ....FKFQ.................................
117.Thunberg         ....MKFQ.................................
203.Paris            ....MKFQ.................................
70.Lily              ....VKFQ.................................
111.Sweet            .........................................
211.Platycodon       ....MKFQ.................................
124.Tulip            .........................................
167.Donkey           ....THQ..................................
73.Moroccan          ....VYHQ.................................
179.Sudan            ....VYHQ.................................
14.Algerian          ....VYHQ.................................
197.wild             ....VYHQ.................................
137.Zucchini         ....VYHQ.................................
84.Papaya            ....VYHQ.................................
```

209.Zucchini	....AYHQ..................................
79.Ornithogalum	....VYHQ..................................
190.Gladiolus	....VYHQ..................................
202.Ornithogalum	....VYHQ..................................
126.Vallota	....VYHQ..................................
80.Ornithogalum	....VYHQ..................................
95.Pepper	....VRHQ..................................
201.Tobacco	....VRHQ..................................
101.Potato	....VRHQ..................................
15.Alstroemeria	....VRHQ..................................
32.Bramble	....VRHQ..................................
173.Bidens	....VRHQ..................................
92.Pepper	....VRHDTNVRHQ............................
109.Sunflower	....VRHQ..................................
192.Sunflower	....VRHQ..................................
16.Potyvirus	....VRHQ..................................
171.Barbacena	....VRHQ..................................
188.Tomato	....VRHQ..................................
189.Pennisetum	....VRHQ..................................
20.Arracacha	....VEHQ..................................
21.Arracacha	....VEHQ..................................
129.Verbena	....VHHQ..................................
222.Verbena	....VHHQ..................................
174.Brugmansia	....VHHQ..................................
220.Mashua	....VRHQ..................................
30.Bidens	....VRHQ..................................
17.Amazon	....VQHQ..................................
96.Peru	....VRHQ..................................
131.Wild	....VRHQ..................................
100.Potato	....VRHQ..................................
91.Pepper	....VRHQ..................................
33.Brugmansia	....VRHQ..................................
54.Gloriosa	....
120.Tobacco	....VRFQ..................................
50.Endive	....VRHQ..................................
110.Sweet	....VYHQ..................................
114.Sweet	....VYHQ..................................
113.Sweet	....VYHQ..................................
187.Sweet	....VYHQ..................................
115.Sweet	....VYHQ..................................
36.Carrot	....
99.Potato	....VQFQ..................................
170.Tamarillo	....VQFQ..................................
221.Potato	....VHFQ..................................
194.Jasmine	....VHFQ..................................
72.Malva	....VYHQ..................................
103.Scallion	....VYHQ..................................
125.Turnip	....VYHQ..................................
66.Kalanchoe	....VYHQ..................................
11.Plum	....VHHQ..................................
215.Scorzonera	....
127.Vanilla	....VFHQ..................................
60.Hyacinth	....VKHQALDDIKDEFSERNLHVDFELDGDVVPGNMSIDHT
19.Apium	....VYHQ..................................
37.Carrot	....VYHQ..................................
39.Celery	....VYHQ..................................
82.Panax	....VFHQ..................................
75.Narcissus	....VYHQ..................................
76.Narcissus	....VYHQ..................................
168.Wild	....VYHQ..................................
64.Japanese	....VYHQ..................................
175.Callistephus	....VYHQ..................................
40.Chilli	....VVHQ..................................
119.Tobacco	....VIHQ..................................
55.Habenaria	....VNHQ..................................
62.Iris	....VNHQ..................................
57.Henbane	....
93.Pepper	....VNHQS.................................
94.Pepper	....VNHQ..................................
41.Chilli	....
132.Wild	....VNHQ..................................
83.Papaya	....VAHQ..................................
219.Thladiantha	....VAHQ..................................
135.Yam	....VAHQ..................................
122.Tuberose	....VHHQ..................................
123.Tuberose	....VHHQ..................................
67.Konjac	....VRHQ..................................
51.Euphorbia	....TKHQ..................................
200.Noni	....VKHE..................................
38.Catharanthus	....VYHQ..................................
176.pecan	....MTFQ..................................
69.Lettuce	....VRHQ..................................
102.Ranunculus	....VYHQ..................................
186.Dioscorea	....VIHQ..................................
196.lupine	....VYHQ..................................
46.Daphne	....VQYQ..................................
47.Daphne	....VKYQ..................................
218.Thevetia	....VDYQ..................................
161.Blackberry	NPFNIIFQ..................................
10.Passiflora	....VVFE..................................
2.Wheat	....CEYQ..................................
3.Oat	....CEYQ..................................
4.Tall	....CEYQ..................................
6.Yellow	....AQYQ..................................
5.Wheat	....CEFQ..................................
1.brome	....SIYQ..................................
140.Sugarcane	PE.SLVFHA.................................
141.Triticum	SD.SFVFHA.................................
139.Caladenia	NN.HITYQ..................................
151.Cassava	...FIERQ..................................
155.Ugandan	...FIEKQ..................................
152.Cucumber	...LIELQ..................................
153.Squash	...IVELQ..................................

```
112.Sweet           ...GIE.....................................
142.Artichoke       ...TSGWLVG.....GTATEELQMHIFDV.............
148.Narcissus       ...TSGWIMG.....GTATEELQ...................
145.Chinese         ...TTGWLTS.....GGGTQELQ...................
150.Yam             ...TTGWLTS.....GGGTQQLQ...................
149.Yam             ...TSGWLVG.....GANTQELQ...................
146.Large           ...TSGWIMK.....GEGTQELQMKLFDL.............
147.Macluravirus    ...TSGWMMG.....GTGTQELQMELFDL.............
144.Cardamom        ...TAGWVMR.....GNDSQELQ...................
143.Broad-leafed    ...TTGWLVG.....G..........................
162.Bellflower      ...TRNILPR.....IINGTVLQ...................
159.Wheat           ...HQGIAS....RMLSASYGLQ...................
160.Wheat           ...HQGIAS....RMFSASYGLQ...................
157.Barley          ...HQGIAS....RMFGASYGLQ...................
158.Oat             ...YQGIAS....KLTQATYGLQ...................
163.Areca           ..........................................
164.Areca           ..........................................
156.Barley          ...NRGFATPALDNLTDFTTILQ...................
154.Tomato          R...VYIQ..................................
```
